# Genome Analysis and Genetic Stability of the *Cryptophlebia leucotreta* Granulovirus (CrleGV-SA) after 15 Years of Commercial Use as a Biopesticide

**DOI:** 10.3390/ijms18112327

**Published:** 2017-11-03

**Authors:** Marcel van der Merwe, Michael D. Jukes, Lukasz Rabalski, Caroline Knox, John K. Opoku-Debrah, Sean D. Moore, Martyna Krejmer-Rabalska, Boguslaw Szewczyk, Martin P. Hill

**Affiliations:** 1 Department of Biochemistry and Microbiology, P.O. Box 94, Rhodes University, Grahamstown 6140, South Africa; vandermerwe_marcel@yahoo.com (M.v.d.M.); mrmdjukes@gmail.com (M.D.J.); caroline.knox@ru.ac.za (C.K.); 2 Department of Recombinant Vaccines, Intercollegiate Faculty of Biotechnology University of Gdansk and Medical University of Gdansk, 80-210 Gdansk, Poland; martyna.krejmer@biotech.ug.edu.pl (M.K.-R.); boguslaw.szewczyk@biotech.ug.edu.pl (B.S.); 3 River Bioscience, P.O. Box 20388, Humewood, Port Elizabeth 6013, South Africa; john@riverbio.com; 4 Citrus Research International, P.O. Box 20285, Humewood, Port Elizabeth 6013, South Africa; seanmoore@cri.co.za; 5 Department of Zoology and Entomology, P.O. Box 94, Rhodes University, Grahamstown 6140, South Africa; m.hill@ru.ac.za

**Keywords:** betabaculovirus, false codling moth, biopesticide, genome stability

## Abstract

*Thaumatotibia leucotreta* Meyrick (Lepidoptera: Tortricidae) is an indigenous pest in southern Africa which attacks citrus fruits and other crops. To control *T. leucotreta* in South Africa, an integrated pest management (IPM) programme incorporating the baculovirus *Cryptophlebia leucotreta* granulovirus (CrleGV-SA) as a biopesticide has been implemented. This study investigated the genetic stability of a commercially produced CrleGV-SA product that has been applied in the field since 2000. Seven representative full-genome sequences of the CrleGV-SA isolate spanning a 15-year period were generated and compared with one another. Several open reading frames (ORFs) were identified to have acquired single nucleotide polymorphisms (SNPs) during the 15-year period, with three patterns observed and referred to as “stable”, “reversion”, and “unstable switching”. Three insertion events were also identified, two of which occurred within ORFs. Pairwise multiple alignments of these sequences showed an identity ranging from 99.98% to 99.99%. Concentration-response bioassays comparing samples of CrleGV-SA from 2000 and 2015 showed an increase in virulence toward neonate *T. leucotreta* larvae. The CrleGV-SA genome sequence generated from the 2015 sample was compared to the Cape Verde reference genome, CrleGV-CV3. Several fusion events were identified between ORFs within these genomes. These sequences shared 96.7% pairwise identity, confirming that CrleGV-SA is a genetically distinct isolate. The results of this study indicate that the genome of CrleGV-SA has remained stable over many years, with implications for its continued use as a biopesticide in the field. Furthermore, the study describes the first complete baculovirus genome to be sequenced with the MinION (Oxford Nanopore, Oxford, UK) platform and the first complete genome sequence of the South African CrleGV isolate.

## 1. Introduction

*Thaumatotibia leucotreta* Meyrick (Lepidoptera: Tortricidae), commonly known as false codling moth, is indigenous to sub-Saharan Africa and an important pest for the African citrus industry [1]. The pest is not only capable of causing economic losses in the field, but postharvest infestation can affect market acceptability of the fruits [1,2]. To control *T. leucotreta* in South Africa, an integrated pest management (IPM) programme involving a wide range of control measures, including insecticides, mating disruption, sterile insect technique (SIT), and biopesticides has been implemented [3,4]. One important component of this control programme is the *Cryptophlebia leucotreta* granulovirus (CrleGV). Angelini and colleagues [5] were the first to describe CrleGV in infected *T. leucotreta* larvae in the Ivory Coast. Additional isolates were subsequently recovered from infected larvae on the Cape Verde Islands and from laboratory-reared insects collected in South Africa and housed at the Hoechst Corporation in Germany [6,7]. Restriction enzyme analysis of genomic DNA revealed that all three isolates were distinct genotypes, and a full genome sequence of the CrleGV Cape Verde 3 (CrleGV-CV3) isolate was subsequently obtained and deposited in the NCBI’s GenBank (Accession number NC_005068) [8,9]. To date, this genome sequence is the only one available for comparison with novel and existing CrleGV isolates and thus serves as a reference isolate.

The South African isolate, CrleGV-SA, was genetically characterised by Singh et al. [10]. Following the development of a virus production system and extensive field trials, the virus was formulated and registered as the biopesticide Cryptogran (River Bioscience (Pty) Ltd., Port Elizabeth, South Africa), and has been applied successfully in the field for more than 10 years [11].

The effectiveness of CrleGV-SA as a biopesticide may be influenced by, among other things, the biological activity or virulence of the virus isolate, which often consists of a mixture of genotypes used in a particular product [11,12]. Baculoviruses are known to exist as genotypic mixtures consisting of different genotypes of the same virus because of host interactions and ecological factors [13,14]. It has been reported that different genotypes can differ in virulence against host insects, thereby influencing the effectiveness of the biopesticide [15,16]. Additionally, genetic variation in field populations of baculoviruses, which involve mutations or recombination events specifically within genes related to oral susceptibility, can have a significant effect on virulence [17,18,19]. Thus, genome stability is an important aspect of a virus’s biological activity that requires investigation, especially when a biopesticide such as CrleGV is frequently applied in the field over many years. 

The aim of this study was to investigate the genetic stability of CrleGV-SA over a 15-year period by complete genome sequencing and analysis of samples collected between 2000 and 2015. A comparison of the biological activity of CrleGV-SA produced in 2000 and 2015 was also performed. Lastly, the complete genome sequence of CrleGV-SA from 2015 was compared to that of CrleGV-CV3 which was previously described and annotated by Lange and Jehle [9].

## 2. Results

### 2.1. Comparison of CrleGV-SA Genome Sequences Across a 15-Year Period

Seven complete genome sequences of CrleGV-SA spanning a 15-year period were generated and compared by multiple alignments. Analysis of these genomes from 2000, 2003, 2005, 2007, 2009, 2012, and 2015 revealed a high degree of genome stability. Of the 127 ORFs present in the CrleGV-SA genome, 116 (91.3%) were identical in terms of nucleotide sequences. The remaining nine ORFs varied in their sequences across this period, with three distinct patterns observed (Table 1). Firstly, in ORFs 81, 109, and 117 a “stable-change” pattern was observed where, once the change had occurred, it was maintained in samples in subsequent years (e.g., A → T). In ORF 81, a synonymous SNP was observed at position 70586, changing the codon from a thymine (T) to a cytosine (C) in the 2007–2009 period. A non-synonymous SNP was observed in ORF 109, whereby an adenine (A) changed to a guanine (G) at position 94106 in the 2003–2005 period. A stable non-synonymous SNP was detected at position 101134 in ORF 117 and resulted in an A changing to a G in the 2007–2012 period. This nucleotide was represented by the ambiguous nucleotide purine (R) (either A or G) in the 2009 sequence. 

The second pattern, a “reversion”, was observed in ORFs 45 and 119. This second pattern was characterised by an SNP which later reverted to its original nucleotide (e.g., A → T → A). A synonymous SNP was observed in ORF 45, whereby a T was replaced by a C before reverting to a T at position 38194. This SNP was observed in the 2012 sequence and was determined to be a T in all subsequent years. The other reverting SNP was observed in ORF 119, whereby a G changed into an A before reverting to a G at position 104415. For this ORF, the SNP occurred across two samples, initially represented as an R in the 2009 sample, then identified as an A in the 2012 sample, and finally reverting to a G in the 2015 sample.

The third pattern observed was termed an “unstable switch”, whereby, as time progressed, ORF sequences changed from an original state to a mutated state. This was then followed by a reversion to the original state, before once again switching to the mutated state (e.g., A → T → A → T). This pattern was observed in ORFs 94 and 120. A non-synonymous SNP was observed in ORF 94, whereby the nucleotide at position 79955 was first determined to be a G in the 2000, 2003, and 2005 sequences, before changing to an R in the 2007 and 2009 sequences. This SNP then reverted to a G in the 2012 sequence, before finally being replaced by an A in the 2015 sample. Similarly, an SNP was observed at position 104594 in ORF 120, which changed from a C to a pyrimidine (Y), then to a C, and lastly to a T in the 2007, 2009, 2012, and 2015 sequences, respectively.

Additional SNPs were observed in non-coding regions, with eight “stable changes” and one “unstable switch” observed. The last major change observed between 2000 and 2015 was the insertion of several nucleotides in three regions. The first of these occurred in ORF 46, involving an insertion of four nucleotides between positions 39147 and 39150, and was observed in the 2005 and 2007 sequences, resulting in a frame shift and shortening of this ORF. The second insertion was observed in the 2007 sequence, having no effect on the amino acid sequence for this ORF. For both these ORFs, these insertions were not observed in sequences from the following years. A small insertion of two nucleotides was observed at positions 75271 and 75272 in the 2005 sequence, which was not observed in sequences from the following years. 

### 2.2. Biological Activity of CrleGV-SA 2000 and 2015

**Figure 1:**
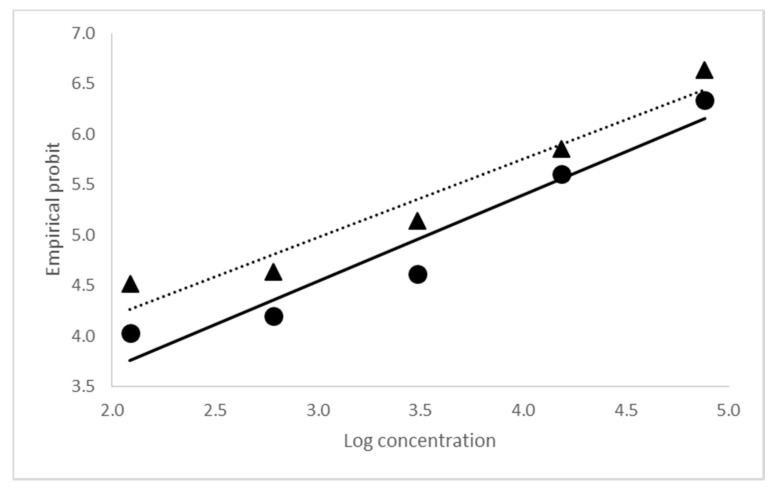
Concentration-response probit lines for CrleGV-SA produced in 2000 (circles and solid line) and 2015 (triangles and dotted line) against neonate *T. leucotreta* larvae.

The biological activity or virulence of CrleGV-SA produced in 2000 and 2015 were determined and compared. Overall, the mean concentrations that would kill 50% and 90% of individuals in a sample (LC_50_ and LC_90_) for the three samples from 2000 and the three samples from 2015 were calculated as 4.071 × 10^3^ and 9.590 × 10^4^ occlusion bodies per mL (OBs/mL) (χ^2^ = 11.644; df = 3; *p* = 0.007), and 1.170 × 10^3^ and 7.849 × 10^4^ OBs/mL (χ^2^ = 11.429; df = 3; *p* = 0.011), respectively (Table 2). The slopes of the two probit lines, 2000 and 2015 (Figure 1), were compared and found to be parallel (χ^2^ = 3.449; df = 1; *p* = 0.06), thus their elevations could be compared. Interestingly, the elevations were found to be significantly different (F_1,7_ = 6.169; *p* = 0.042), as a result of the higher mortality recorded in 2015. Despite this, differences in the concentration-response results measured in 2000 and 2015 were not great, and it could be concluded that there was no reduction in virulence over time. Additionally, this difference may even have been a result of the higher control mortality recorded in the bioassays for the 2000 sample (12%), compared to the 2015 sample (5%).

### 2.3. Comparison of the CrleGV-SA 2015 Genome Sequence to CrleGV-CV3

**Figure 2:**
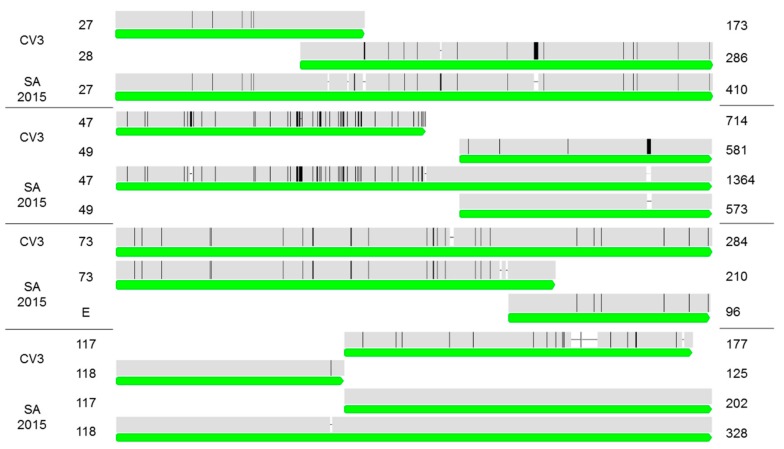
Nucleotide alignments of fusion events in CrleGV-CV3 and CrleGV-SA (2015) for ORFs 27/28, 47/49, 73/E, and 117/118. Green bars show gene annotations, with the graphs highlighting disagreements as black bars in each alignment to the consensus sequence. Gaps in each alignment are shown as white spaces in the graphs, with the amino acid length of each ORF shown to the right.

A total of 133 ORFs were identified within the 111,334 bp genome of CrleGV-SA (2015). Comparison with the CrleGV-CV3 genome showed an overall pairwise identity of 96.6% and an AT content of 67.4%, with 127 of the reference ORFs identified. Additionally, three fusion events were identified within the CrleGV-SA genome when compared with CrleGV-CV3, involving ORFs 27/28, 47/49, and 117/118 (Figure 2). ORFs 49 and 117 continued to exist alongside the fused ORFs 47 and 118 in CrleGV-SA. However, ORF 28 identified in CrleGV-CV3, was disrupted by the fusion event, leading to a premature stop codon and truncation of the ORF. In addition, ORF 73 in CrleGV-CV3 appeared as two distinct ORFs in CrleGV-SA, namely, a truncated ORF 73 and a newly identified ORF E (Figure 2). Interestingly, CrleGV-SA ORF 48 was observed to have a truncation of 104 amino acids, while a mutation within ORF 126 similarly resulted in a severe truncation of 28 amino acids. The analysis of the six additional ORFs (ORFs A to F) identified in the SA genome indicated that there were four ORFs (A, B, D, and E) that appeared to be non-putative in nature, with potential homologs identified, albeit with very low percentage identities (32–43%). Only the newly identified ORF E in CrleGV-SA showed homology towards a known baculovirus protein, ORF 82, in *Cydia pomonella* granulovirus (CpGV). However, this was expected, as CrleGV-CV3 ORF 73 was previously identified as being similar to this ORF in CpGV.

**Figure 3:**
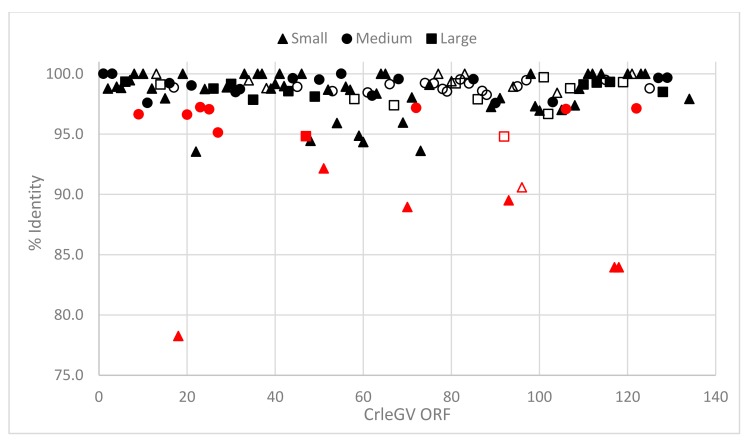
Scatter graph of the percentage identity between ORFs in CrleGV-SA (2015) and CrleGV-CV3, grouped by amino acid length. Triangles are ORFs < 200 AA, circles are ORFs > 200 but < 400 AA, and squares are ORFs > 400 AA. Core genes are shown with shape outlines only, while outliers for each group are coloured red.

Further analysis of the CrleGV-SA ORFs revealed sixteen outliers (shown in red in Figure 3), based on the standard deviation observed in the percentage identity for each group when compared to the CrleGV-CV3 isolate. While many of the outliers were observed to be hypothetical proteins, several were matched in the reference genome, including ac78, tlp20, iap-5, dbp, pe-38, and vp-91, and were identified as ORFs 96, 93, 106, 72, 25, and 92 respectively. Three of the fused ORFs were also identified as outliers when compared to their respective counterparts in the reference genome. Only ORF 13, which was identified as odv-e18, was found to remain stable throughout the 15-year period, while also maintaining an identical sequence to the corresponding ORF in the CrleGV-CV3 isolate.

## 3. Discussion

To examine the genetic stability of CrleGV-SA over time, complete genome sequences were obtained across a period spanning from 2000 to 2015, and their nucleotide sequences compared. The CrleGV-SA genome was observed to be highly stable across this period with only a few ORFs changing. Interestingly, three patterns of change were observed. The first, referred to as a “stable change”, showed that the occurrence of SNPs in ORFs was transmitted into future samples of the virus. The second observed pattern of change was referred to as a “reversion”, whereby an SNP in an ORF reverted to its original nucleotide. The third observed pattern of change, and possibly the most interesting, was the “unstable switching” pattern, whereby SNPs would alternate between the original and the modified sequence. In each of the ORFs where this pattern was observed, the most recent sample (2015) contained the mutated ORF sequence, thus making it unclear whether the mutation would ultimately become stable or undergo another reversion. When considering these SNP patterns, it is important to note that the samples of CrleGV-SA which were chosen for sequence analysis likely comprise several genotypes, as the original inoculum was obtained from field collected insects. Mixed genotypes are well described in baculoviruses (see for example [13,14,17]), with the SNPs reported in this study likely representing the most dominant in them. In instances where there is no significant difference between the ratio of these genotypes, SNPs would appear as ambiguous nucleotides, and the ambiguity reported was observed to accommodate either of the nucleotides determined in the year before and after the change. For example, the SNP in ORF 117 was first identified as an A before being replaced with a G, with the intermediate ambiguous nucleotide (R) which represents either an A or G. In general, it was expected that there would be very little genetic variation in CrleGV-SA genomes over the 15-year period, as all samples were taken from a single production entity. For example, when, in the early 1980s, commercial production and application on soya of the *Anticarsia gemmatalis* nucleopolyhedrovirus (AgNPV) began against the velvet bean caterpillar in Brazil [20], it was possible to produce aliquots of the original isolate for subsequent large scale production, thus maintaining genetic integrity and avoiding loss of virulence [21]. However, as the program progressed, this was no more possible because of the large amounts of virus required for AgNPV field production. Consequently, AgNPV was produced in the field from the inoculum multiplied in the previous season. The analysis of AgNPV isolates showed that the virus varied in certain locations of the genome, indicating that the AgNPV had changed genetically in relation to the original wild isolate [22]. However, despite this, the genomic analysis of the AgNPV variants obtained from field multiplication during many years indicated that the virus maintained considerable stability [21]. Bioassays or field tests with batches of AgNPV, obtained each year from field-collected larvae from 1979 to 1995, showed that AgNPV virulence toward the host was not altered significantly [23,24].

The pre-commercial or research-phase production of CrleGV (in 2000) was conducted by Citrus Research International, and the commercial production (2000–2015) was conducted by River Bioscience. Consistency in isolate management was maintained from the research to the commercial phase. However, as reported by Grzywacz and Moore, a stock suspension of the chosen isolate was prepared as an inoculum to produce all future batches of virus [12]. Nevertheless, it was still possible that some genetic change in the isolate could have taken place, as it was necessary to periodically passage the inoculum through host larvae to amplify the inoculum stock for production.

Regarding the biological activity, genotypic variations between virus isolates of the same species have been recorded in laboratory bioassays and may be associated with phenotypic variations such as virulence [25]. Minor genetic changes in the form of SNPs were observed over the period of analysis, but whether these are associated with changes in phenotypic traits still needs to be investigated. Although it may be considered a positive outcome that the virulence obtained for the 2015 samples was higher than that of the 2000 samples, albeit only a 3.5-fold and a 1.5-fold difference in LC_50_ and LC_90_, respectively, this difference does require an explanation. A possible reason could be related to the source of the insect host material, which was initially heterogeneous in nature during its early years of establishment, when there was periodic replenishment of the culture with wild moths [2]. However, over time and during the entire 15-year period, without the frequent or continuous replenishment of these mixed insect populations in the laboratory, the insects may have started to become more genetically homogenous in nature, thereby leading to individuals that were more susceptible to the virus, as seen with the 2015 samples. Opoku-Debrah et al. (2016) also showed that virulence is a host-virus relationship [16], rather than a simple phenotypic characteristic of the virus. Consequently, any change in the genetic material of the host against which the bioassays are conducted could have phenotypic implications, which may result in a difference in virulence, even if there is no change in the virus. 

Another primary objective of this study was to obtain a complete genome sequence of CrleGV-SA and compare this to a reference isolate, CrleGV-CV3, currently the only CrleGV genome available for comparison of different isolates [9]. The use of two next generation sequencing (NGS) approaches in this study, namely Illumina and Nanopore, to assemble the CrleGV-SA (2015) genome confers a high degree of accuracy on the assembled genome. Furthermore, this sequence represents the first baculovirus genome to be fully assembled using Nanopore technology, more specifically the MinION device. While these NGS platforms use completely distinct molecular principles to obtain sequence reads, complete agreement was achieved between the assembled genomes, and further accuracy was achieved by combining the sequence data into a single assembly using the SPAdes genome assembler. Illumina platforms provide a high degree of mutation detection confidence because of the large amount of reads produced, concomitantly with a low degree of genome structure confidence, which results from the short length of these reads. Conversely, Nanopore platforms provide a lower degree of mutation detection confidence and a high degree of structure confidence, as a result of the longer, albeit fewer, reads. Thus, combining these technologies offers the potential to obtain both high mutation detection and increased structural confidence [26].

This increased level of assembly accuracy revealed differences between the reference genome and that of the South African isolate. The most significant of these was the identification of four fusion events, and the truncation and deletion of ORF 47 and 126, respectively. Among the ORFs identified, CrleGV-SA (2015) ORF 10, a potential chitinase encoding gene, matched CrleGV-CV3 ORF 10 and appeared to be truncated and non-functional, as discussed by Lange and Jehle [9]. The truncation of this gene in both isolates may indicate that this event occurred early in the evolution and diversification of CrleGV from other baculoviruses. Furthermore, Lange & Jehle identified five ORFs which appeared to be non-coding because of the presence of homologous repeat sequence patterns (*hrs*). Three of the four novel ORFs identified (A, B, and D) in the SA genome fall within these *hrs* regions and are therefore likely to be the same non-coding ORFs previously identified in CrleGV-CV3. The fourth ORF E identified does not appear within any *hrs* regions and may be a novel ORF in the SA isolate, i.e., a result of a splicing/fusion event of ORF 73 in CrleGV-CV3 into two separate ORFs in CrleGV-SA. Excluding the three non-functional (10, 48, and 126), the two putative (C and F), and the three non-coding (A, B, and D) ORFs, a total of 127 ORFs are present in the CrleGV-SA (2015) genome compared to 129 ORFs in the CrleGV-CV3 genome. Nucleotide sequence variation between CrleGV-SA (2015) and CrleGV-CV3 was observed in almost every ORF, except for odv-e18 which was identical in these isolates. However, when comparing amino acid sequences, the number of identical ORFs in these isolates increased to 22, indicating that many of the nucleotide changes observed are synonymous mutations. These results, as well as gene sequence comparisons between the CrleGV-SA and the Cape Verde isolates, provide further support for the observation that different CrleGV isolates exhibit genetic variation, as reported by Opoku-Debrah et al. [14].

The results of this study indicate that, with respect to the genetic composition determined by the incidence of SNPs and the virulence of the 2000 and 2015 samples evaluated through biological assays, both the genotypic and phenotypic integrity of the commercially produced CrleGV-SA have remained relatively consistent over a 15-year period, thus confirming the continued suitability of this virus as a biopesticide for the control of *T. leucotreta* in the field. If any change in the efficacy of a commercially used CrleGV isolate were to be observed in the field, it could at least be concluded that the problem is unlikely to lie within the integrity of the virus, if consistent production methods are maintained. Rather, other factors related to the management of the virus products, such as the timing of application and the application efficacy, could be investigated. Alternatively, the potential development of resistance in target pests towards isolates because of the continuous biopesticide application in the field, as it was seen with CpGV in Europe against *Cydia pomonella* [27], may also warrant investigations, as well as support, for continued research and bioprospecting for novel isolates which can be formulated into commercial biopesticides. 

## 4. Materials and Methods

### 4.1. Host Material

Host insects were sourced from a heterogeneous *T. leucotreta* culture initially established by Citrus Research International (CRI), Port Elizabeth, South Africa [28]. This population was established on artificial diet in the laboratory, using field collected *T. leucotreta* larvae, isolated from infested citrus fruits (oranges), collected from Citrusdal (Western Cape), Zebediela (Limpopo), and Addo (Eastern Cape) in South Africa [14,28]. Furthermore, periodic replenishment of the culture with wild moths was performed across the 15-year period, when necessary.

### 4.2. Virus Inoculum

CrleGV-SA was originally obtained from diseased larvae in the culture [29]. Once production of CrleGV-SA was initiated, originally for research purposes, a stock suspension of the virus was prepared and used for production of all future batches [12,28]. Apart from being used for virus production, the stock suspension was passaged through fourth and fifth instar larvae to amplify the original stock suspension. Samples of CrleGV-SA were acquired from *T. leucotreta* larval homogenate samples produced in 2000, 2003, 2004, 2007, 2009, and 2012. The 2015 CrleGV-SA sample was purified from *T. leucotreta* larvae infected with CrleGV-SA in 2015. The homogenate samples were stored at −20 °C by Citrus Research International (Port Elizabeth, South Africa) and were supplied alongside the 2015 infected larval samples containing CrleGV-SA, produced by River Bioscience. However, all samples originated from the same *T. leucotreta* culture described above. Purification of the CrleGV-SA (2015) occlusion bodies (OBs) was carried out using a 30–80% glycerol gradient protocol, using approximately 1.8 g of infected insect larval cadavers [28,30]. 

### 4.3. Genomic DNA Extraction

A modified CTAB (Hexadecyltrimethylammonium bromide) DNA extraction protocol described by Opoku-Debrah et al. (2013) was used to extract genomic DNA from OBs [14]. Briefly, a 200 μL sample was mixed with 90 μL of 1 M Na_2_CO_3_ and incubated at 37 °C for 30 min. Samples were treated with 120 μL of 1M Tris-HCl (pH 6.8), 90 μL of 10% SDS and 20 μL of Proteinase K (25 mg/mL), and further incubated at 37 °C for 30 min. A 10 μL volume of RNase A (10 mg/mL) was added, followed by a final incubation at 37 °C for 30 min. Samples were centrifuged at 12,100× *g*, the supernatants were collected, and 400 μL of CTAB, preheated to 70 °C, was added (54 mM CTAB, 0.1 M Tris-HCl pH 8, 20 mM Na_2_EDTA, 1.4 M NaCl) before incubation at 70 °C for 45 min. A volume of 400 μL of chloroform, pre-cooled to 4 °C, was added to the CTAB mixture and inverted several times before centrifugation at 6700× *g*. The upper aqueous phase was collected and 400 μL of ice-cold isopropanol were added to precipitate the DNA overnight at −20 °C. The DNA was pelleted at 12,100× *g* for 20 min and washed with ice-cold ethanol (70% *v*/*v*, −20 °C) prior to a final centrifugation at 12,100× *g* for 5 min. The pellet was left to dry, ensuring all ethanol was removed before re-suspending the samples in 20 μL of ddH_2_O and storing them at −20 °C until use. 

### 4.4. CrleGV-SA Genome Sequencing and Analysis

The complete genomes of multiple CrleGV-SA samples were sequenced using two next generation platforms: MiSeq (Illumina, San Diego, CA, USA) and MinION (Oxford Nanopore, Oxford, UK). CrleGV samples were collected in 2000, 2003, 2005, 2007, 2009, 2012, and 2015 (among others not reported here) and were sequenced using the MiSeq platform. For MiSeq genomic library preparation, the Nextera XT DNA kit (Illumina, San Diego, CA, USA) was used according to the manufacturer’s protocol. During the machine run, paired reads of the target length 2 × 300 bp were generated. The 2015 sample was further sequenced with the MinION platform. Sequencing libraries were also prepared in accordance to the manufacturer’s guidelines using the SQK-NSK007 Nanopore Sequencing Kit (R9) (Oxford Nanopore, Oxford, UK) for the Native Barcode procedure. After the 24 h machine run, long (>1000 nt) 2D-reads were generated. Before library sequencing procedures, all isolated DNA was tested for its purity, concentration, and quality on a TapeStation 2200 (Agilent Technologies, Santa Clara, CA, USA) and a Quantus Fluorometer (Promega, Madison, WI, USA). For further bioinformatics analyses, fastq raw reads data files were generated by BaseSpace Sequence Hub (Illumina) for the MiSeq reads, and Poretools software for MinION reads [31]. Reads were quality-checked, and errors were corrected before assembly using three separate applications (Table S1). First, the Illumina generated reads were assembled de novo using Geneious R9 [32]. Second, the reads generated with the MinION platform were de novo assembled separately using the Canu assembly pipeline [33]. For the 2015 sample, both sets of reads, Illumina and Nanopore, were jointly de novo assembled using the SPAdes genome assembler [34]. Multiple complete CrleGV-SA genome sequences were generated for years 2000, 2003, 2005, 2007, and 2012. A single consensus sequence was generated for these years in Geneious R7 using a strict (>50%) threshold and assigning base pairs with the highest quality. These assemblies were compared for consistency by multiple alignments in Geneious R9. Open reading frames (ORFs) were annotated using two independent approaches: first by comparison with the reference CrleGV-CV3 genome, and secondly by performing an ORF search using the Glimmer software (gene model built on the CrleGV-CV3 genome) [35]. The second approach identified six additional ORFs, each of which encode proteins greater than 50 amino acids in length. The putative nature of these additional ORFs was checked using HMMER (phmmer, UniProtKB) (http://hmmer.org/) [36]. Gene comparisons between the SA sample from 2015 and the CV isolate were performed with the aid of the NCBI pBLAST (https://blast.ncbi.nlm.nih.gov/Blast.cgi), with ORFs binned into three groups—small (<200 AA), medium (>200 but <400 AA), and large (>400 AA)— and percentage identity plots produced for each group. Outliers were determined by calculating the mean percentage identity and the standard deviation for each group. ORFs with percentage identities lower than the mean minus the calculated standard deviation were identified as outliers. The CrleGV-SA genome sequence was submitted to GenBank under accession number MF974563.

### 4.5. Biological Assays 

The virulence of CrleGV-SA from 2000 and 2015 was compared using surface-inoculated, concentration-response bioassays with neonate *T. leucotreta* larvae on artificial diet, as described by Moore et al. (2011), with three replicates performed for each sample [29]. These bioassays were performed at the time the virus samples were recovered. A five-fold serial dilution of CrleGV-SA 2000 or 2015 produced five treatments (1.221 × 10^2^, 6.104 × 10^2^, 3.052 × 10^3^, 1.526 × 10^4^, and 7.630 × 10^4^ OBs/mL). These and an untreated control were used, each with 50 larvae (hence a total of 150 larvae per treatment for the three replicates) placed individually into the cells of the bioassay trays. The larvae were maintained at 27 °C with a relative humidity of approximately 60%. Mortality was evaluated after 7 days, according to standard practice with this particular virus-host combination, and concentration-response curves were calculated using PROBAN, a computer programme used for probit analysis [16,29,37,38]. PROBAN corrected the control mortality according to Abbott’s formula [39]. Three replicates from the dose-response bioassays were pooled together, and their LC_50_ and LC_90_ values determined. PROBAN transformed the doses to log_10_, and the percentage mortality response to empirical probits. Using this information, the fit of the probit (regression) lines were calculated, as were the fiducial limits. For the comparison of slopes (parallelism), Chi-square tests were employed for parallelism, and *F*–tests were used for homoscedasticity. From this, LC_50_ and LC_90_ values were calculated for each assay. Additionally, probit lines of the 2000 and 2015 samples were compared to determine whether significant differences occurred between the slopes of the lines, indicating a difference in virulence.

## Figures and Tables

**Table:** 

Nucleotide Position		21022	21222	22400	22404	22414	22418	22427	22431	22445	38193	38194	38195	39138	39139	39140	39141	39142	39143	39144	39145	39146	39147	39148	39149	39150
ORF											5′	45	3′	3′	46											5′
Gene & Frame		-	-	-	-	-	-	-	-	-		PIF2			HP											
2015	NT	T	G	A	A	A	A	A	A	A	A	T	T	G	T	T	A	A	A	A	T	T	-	-	-	-
	AA											I			N			F			N					
2012	NT	Y	C	A	A	A	A	A	A	A	A	C	T	G	T	T	A	A	A	A	T	T	-	-	-	-
	AA											T			N			F			N					
2009	NT	C	C	A	A	A	A	A	A	A	A	T	T	G	T	T	A	A	A	A	T	T	-	-	-	-
	AA											I			N			F			N					
2007	NT	Y	C	A	A	A	A	A	A	A	A	T	T	G	T	T	A	A	A	A	T	T	C	G	C	G
	AA											I				*			F			E			R	
2005	NT	T	C	T	T	T	T	T	T	T	A	T	T	G	T	T	A	A	A	A	T	T	C	G	C	G
	AA											I				*			F			E			R	
2003	NT	C	C	W	W	W	W	W	W	W	A	T	T	G	T	T	A	A	A	A	T	T	-	-	-	-
	AA											I			N			F			N					
2000	NT	C	C	W	W	W	W	W	W	W	A	T	T	G	T	T	A	A	A	A	T	T	-	-	-	-
	AA											I			N			F			N					

**Table:** 

Nucleotide Position		62164	62165	62166	62167	62168	62169	62170	62171	62172	62173	62174	62175	62176	62177	62178	62179	62180	62181	62182	62183	62184	62185	62186	62187	62188	62189
ORF		5′	75								3′	5′	75														3′
Gene & Frame			HP										HP														
2015	NT	T	A	-	-	-	-	-	-	-	-	-	-	-	-	-	-	T	A	T	A	T	C	G	T	A	G
	AA		Y																	I			S			*	
2012	NT	T	A	-	-	-	-	-	-	-	-	-	-	-	-	-	-	T	A	T	A	T	C	G	T	A	G
	AA		Y																	I			S			*	
2009	NT	T	A	-	-	-	-	-	-	-	-	-	-	-	-	-	-	T	A	T	A	T	C	G	T	A	G
	AA		Y																	I			S			*	
2007	NT	T	A	C	A	T	A	T	C	G	T	A	G	G	T	T	A	T	A	T	A	T	C	G	T	A	G
	AA		Y			I			S			*															
2005	NT	T	A	-	-	-	-	-	-	-	-	-	-	-	-	-	-	T	A	T	A	T	C	G	T	A	G
	AA		Y																	I			S			*	
2003	NT	T	A	-	-	-	-	-	-	-	-	-	-	-	-	-	-	T	A	T	A	T	C	G	T	A	G
	AA		Y																	I			S			*	
2000	NT	T	A	-	-	-	-	-	-	-	-	-	-	-	-	-	-	T	A	T	A	T	C	G	T	A	G
	AA																			I			S			*	

**Table:** 

Nucleotide Position		70584	70585	70586	75271	75272	79953	79954	79955	94105	94106	94107	101133	101134	101135	104415	104416	104417	104593	104594	104595
ORF		5′	81	3′			5′	94	3′	5′	109	3′	3′	117	5′	3′	119	5′	5′	120	3′
Gene & Frame			P33		-	-		AC81			HP			HP			LEF8			HP	
2015	NT	G	A	C	-	-	T	T	A	A	G	A	A	G	T	G	T	T	A	T	G
	AA		D					L			R			T			N			M	
2012	NT	G	A	C	-	-	T	T	G	A	G	A	A	G	T	A	T	T	A	C	G
	AA		D					L			R			T			N			T	
2009	NT	G	A	C	-	-	T	T	R	A	G	A	A	R	T	R	T	T	A	Y	G
	AA		D					L			R			I/T			N			T/M	
2007	NT	G	A	T	-	-	T	T	R	A	G	A	A	A	T	G	T	T	A	C	G
	AA		D					L			R			I			N			-	
2005	NT	G	A	T	C	A	T	T	G	A	G	A	A	A	T	G	T	T	A	C	G
	AA		D					L			R			I			N			-	
2003	NT	G	A	T	-	-	T	T	G	A	A	A	A	A	T	G	T	T	A	C	G
	AA		D					L			K			I			N			-	
2000	NT	G	A	T	-	-	T	T	G	A	A	A	A	A	T	G	T	T	A	C	G
	AA		D					L			K			I			N			-	

NT = nucleotides: Y = C or T; W = A or T; R = A or G; - = gap. AA = amino acids: I = isoleucine; T = threonine; N = asparagine; * = stop codon; F = phenylalanine; E = glutamic acid; R = arginine; S = serine; D = aspartic acid; L = leucine; K = lysine; M = methionine; HP = hypothetical protein, PIF2 = *per os* infectivity factor 2. LEF8 = late expression factor 8.

**Table 2:** Concentration-response values for neonate *T. leucotreta* larvae calculated from three replicates of each of two *Cryptophlebia leucotreta* granulovirus samples produced in 2000 and 2015.

Sample	Lethal Concentration		Standard Error (SE)	95% Fiducial Limits		Means of Empirical Probits	Slope ± SE
				Lower	Upper		
2000	LC_50_	4.071 × 10^3^	± 6.286 × 10^2^	2.974 × 10^3^	5.496 × 10^3^	5.1073	0.9340 ± 0.0765
	LC_90_	9.590 × 10^4^	± 2.702 × 10^4^	5.882 × 10^4^	1.822 × 10^5^		
2015	LC_50_	1.170 × 10^3^	± 2.072 × 10^2^	8.095 × 10^2^	1.636 × 10^3^	5.2293	0.7527 ± 0.0608
	LC_90_	7.849 × 10^4^	± 1.826 × 10^4^	3.455 × 10^4^	1.193 × 10^5^		

## Supplementary Materials

Supplementary materials can be found at http://www.mdpi.com/1422-0067/18/11/2327/s1.
