# Supplementary material for: Genome Analysis and Genetic Stability of the Cryptophlebia leucotreta Granulovirus (CrleGV-SA) after 15 Years of Commercial Use as a Biopesticide
Source: Int J Mol Sci. 2017 Nov 3;18(11):2327. doi: 10.3390/ijms18112327 (PMC5713296; doi:10.3390/ijms18112327)
Supplement: Supplementary file 1 [file ijms-18-02327-s001.pdf]

Table S1. Sequence data used for the assembly of CrleGV-SA genomes for the years 2000, 2003, 2005, 2007, 2009 and 2012 showing the assembly confidence mean, the read quality scores and the number and length of reads used per assembly.

Year	Genome length	Sample	Confidence mean	Q20[1]	Q30[1]	Q40[1]	Error corrected reads	Read length	Expected error
2000	111334	a	79647.1	100	100	100	2547872	50- 301	0
	111334	b	31388	100	100	100	523303	50- 301	0
2003	111334	a	73569.2	100	100	100	2286595	50- 301	0
	111334	b	36397.4	100	100	100	614397	50- 301	0
2005	111329	a	13106.3	100	100	100	396456	50- 301	0.7
	111218	b	991	100	99.9	99.9	16631	50- 251	1.42
2007	111316	a	1342.5	100	99.9	99.9	22013	50- 251	1.07
	111331	b	8526.6	100	100	100	261077	50- 301	0.27
	111350	c	13420.2	100	100	100	161502	50- 301	0
2009	111334	a	10108.6	100	100	100	123787	50- 301	0
2012	110869	a	840	100	100	100	10473	50- 301	0.07
	111334	b	9297.1	100	100	100	112467	50- 301	0

[1]Q = quality score; length in bp (base pair)
